# Encapsulating Peritoneal Sclerosis: Pathophysiology and Current Treatment Options

**DOI:** 10.3390/ijms20225765

**Published:** 2019-11-16

**Authors:** Rajesh M. Jagirdar, Andreas Bozikas, Sotirios G. Zarogiannis, Maria Bartosova, Claus Peter Schmitt, Vassilios Liakopoulos

**Affiliations:** 1 Division of Nephrology and Hypertension, 1st Department of Internal Medicine, Medical School, Aristotle University of Thessaloniki, 54124 Thessaloniki, Greece; raj.jagirdar@gmail.com (R.M.J.); a.bozikas@gmail.com (A.B.); 2 Center for Pediatric and Adolescent Medicine, University Hospital Heidelberg, 69120 Heidelberg, Germany; Sotirios.Zarogiannis@med.uni-heidelberg.de (S.G.Z.); Maria.Bartosova@med.uni-heidelberg.de (M.B.); ClausPeter.Schmitt@med.uni-heidelberg.de (C.P.S.); 3 Department of Physiology, Faculty of Medicine, University of Thessaly, 41500 Larissa, Greece

**Keywords:** encapsulating peritoneal sclerosis, fibrosis, peritoneal dialysis

## Abstract

Encapsulating peritoneal sclerosis (EPS) is a life-threatening complication of long-term peritoneal dialysis (PD), which may even occur after patients have switched to hemodialysis (HD) or undergone kidney transplantation. The incidence of EPS varies across the globe and increases with PD vintage. Causative factors are the chronic exposure to bioincompatible PD solutions, which cause long-term modifications of the peritoneum, a high peritoneal transporter status involving high glucose concentrations, peritonitis episodes, and smoldering peritoneal inflammation. Additional potential causes are predisposing genetic factors and some medications. Clinical symptoms comprise signs of intestinal obstruction and a high peritoneal transporter status with incipient ultrafiltration failure. In radiological, macro-, and microscopic studies, a massively fibrotic and calcified peritoneum enclosed the intestine and parietal wall in such cases. Empirical treatments commonly used are corticosteroids and tamoxifen, which has fibrinolytic properties. Immunosuppressants like azathioprine, mycophenolate mofetil, or mTOR inhibitors may also help with reducing inflammation, fibrin deposition, and collagen synthesis and maturation. In animal studies, N-acetylcysteine, colchicine, rosiglitazone, thalidomide, and renin-angiotensin system (RAS) inhibitors yielded promising results. Surgical treatment has mainly been performed in severe cases of intestinal obstruction, with varying results. Mortality rates are still 25–55% in adults and about 14% in children. To reduce the incidence of EPS and improve the outcome of this devastating complication of chronic PD, vigorous consideration of the risk factors, early diagnosis, and timely discontinuation of PD and therapeutic interventions are mandatory, even though these are merely based on empirical evidence.

## 1. Introduction

Peritoneal dialysis (PD) is a widely used renal replacement therapy allowing end stage renal disease (ESRD) patients to undergo a home-based treatment with significant quality of life benefits. PD uses the peritoneal membrane (PM) as a dialyzing membrane where the exchange of water and solutes takes place [1]. A major shortcoming of PD is the inevitable progressive transformation of the peritoneum with long-term PD. Signs of peritoneal fibrosis are detected in 50% to 80% of patients within one to two years on PD [2]. Even with a neutral pH, low glucose degradation products (GDP) fluids, early angiogenesis, and slowly progressive peritoneal fibrosis develops in the majority of patients [3]. In severe cases, a critical and life-threatening complication may develop known as encapsulating peritoneal sclerosis (EPS). EPS is characterized by ultrafiltration failure (UFF) together with a high transporter status; intestinal obstruction resulting from persistent, major intraabdominal inflammation; encapsulation of the bowels; and by malnutrition and failure to thrive. EPS as an entity was first described in 1980 on a patient in intermittent PD and the first description on continuous ambulatory PD (CAPD) followed 3 years later [4,5].

EPS may also develop in patients with autoimmune diseases, peritoneal and intra-abdominal malignancies, chronic peritoneal ascites, intra-peritoneal chemotherapy, intraperitoneal exposure to particulate matter or a disinfectant, abdominal surgery, endometriosis, and intra-peritoneal infections (tuberculosis) [6,7,8]. These pathomechanisms will not be addressed in this review.

## 2. Epidemiology

The frequency of EPS varies across the globe between 0.5% to 7.3% [6,9,10,11,12,13,14,15], but may be as high as 17.2% in patients undergoing PD for 15 or more years [11]. In a recent study with more than 17,300 patients from Australia, New Zealand, and Scotland, EPS risk increased to 8% after 9 years of PD but was only 1.5% when the competing risk of death was taken into account [16]. The clinical presentation and diagnosis of EPS differ among centers, countries, and over time. EPS may even develop after PD discontinuation, i.e., in patients who have switched to hemodialysis (HD) or have undergone kidney transplantation and are lacking the peritoneal rinsing effect of repeated PD exchanges [17]. In a survey in 14 European pediatric dialysis centers comprising 10 years of retrospective follow-up, EPS prevalence was 1.5% after a median PD vintage of 5.9 (1.6–10.2) years [18].

## 3. Pathophysiology and Risk Factors

### 3.1. Pathophysiology

**Figure 1:**
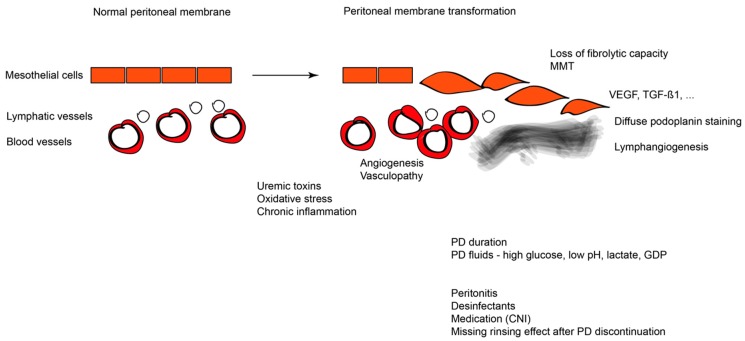
Overview of EPS pathogenesis depicting the normal peritoneum on the left and the changes induced during EPS along with the factors that are involved in EPS on the right. CNI: Calcineurin inhibitors; GDP: glucose degradation products; MMT: mesothelial-to-mesenchymal transition; PD: Peritoneal Dialysis; TGF-β1: Transforming Growth Factor β1; VEGF: Vascular Endothelial Growth Factor.

The pathogenesis of EPS is multifactorial with various initiating and accelerating factors at different stages of the disease [7] (Figure 1). The unanimously recognized risk factor for EPS is long-term PD [19,20,21,22,23,24,25,26,27,28,29]. There is a strong linear correlation between PD duration and the frequency of EPS. PD itself is the first hit leading to a PM transformation [30]. Long-term exposure to PD fluid results in morphologic and functional changes of the PM due to the unphysiological composition of the PD fluids [3,30,31]. Exposure to bioincompatible PD solutions that contain supraphysiological concentrations of glucose, glucose-degradation products (GDP), lactate as a buffer, and having an acidic pH progressively transform the peritoneum, i.e., result in peritoneal mesothelial cell loss, submesothelial fibrosis, and vasculopathy [12,26,29,32,33]. GDP confer significant local and presumably systemic toxicity and give rise to the formation of toxic advanced glycation end products (AGEs) [34,35]. Chronic uremia associated pro-inflammatory and oxidative stress may further accelerate these pathomechanisms. Distinct peritoneal inflammatory changes and slight peritoneal thickening can already be observed in patients with CKD stage 5 [3] and in patients on hemodialysis, respectively [32,36]. Improvement of the manufacturing process, i.e., the introduction of double chamber PD fluids, separating the glucose at a very low pH from the buffer compound, significantly reduced the fluid GDP content and allowed for the introduction of a physiological buffer, namely bicarbonate. In vitro and experimental in vivo studies suggest less peritoneal toxicity [37], but even low GDP fluids with a neutral to physiological pH confer major peritoneal toxicity, and within a few months of PD, induce profibrogenic cytokines, such as transforming growth factor β 1 (TGF-β1), and angiogenic factors, such as vascular endothelial growth factor (VEGF) and endothelial nitric oxide synthase (eNOS) [38], altogether resulting in excessive fibrosis and angiogenesis [21,28]. Mesothelial cells lining the peritoneal cavity are primarily exposed to toxic PD fluids. Mesothelial cells are either detached with chronic PD, resulting in progressive peritoneal denudation, or undergo a mesothelial to mesenchymal transition (MMT), i.e., lose their polarized cytoskeletal organization and cell-to-cell contacts, acquire a myofibroblast-like phenotype with high motility [39], and secrete extracellular matrix compounds and profibrotic and angiogenetic cytokines [40,41,42,43]. In addition, the fibrolytic action of mesothelial cells is lost [44].

In peritoneal biopsies of patients with EPS, there is a loss of normal mesothelial cells, massive expansion of the submesothelial compact zone through the MMT and increased vascularization, mononuclear cell infiltration [45,46,47], calcifications, hyaluronic acid homeostasis disruption [48,49], and a lower peritoneal mast cell number and fibrolytic tryptase abundance as compared to other peritoneal inflammatory conditions, suggesting deficient counter-regulatory actions [50]. The histological hallmark is the abnormal podoplanin staining pattern. Podoplanin (D2–40) is a 38–43 kD mucoprotein, expressed in lymphatic endothelial and in mesothelial cells. Podoplanin expression beyond lymphatic endothelia was detected in pediatric peritoneal biopsies from patients treated using low GDP PD fluids and was associated with more severe peritoneal inflammation, MMT, and vasculopathy [3]. In adult EPS biopsies, podoplanin was co-localized with alpha smooth muscle actin in fibroblast-like cells accumulating in the peritoneal submesothelium [51]. In this study, depending on the morphology of podoplanin staining, patients were grouped according to “low,” “organized,” “diffuse,” and “mixed” podoplanin patterns. Patients with the “low” or “organized” patterns had a significantly lower level of CRP compared to the “mixed” type and thus such morphological stratification might have a diagnostic and prognostic effect [52]. These different morphological groups were frequently present in patients with classical EPS and EPS that developed post transplantation [53]. Beyond podoplanin, connective tissue growth factor 2 (CCN2) was significantly more abundant in the biopsies of EPS patients [54] and can be blocked by a monoclonal antibody FG-3019 [55], possibly representing a therapeutic target.

These morphological alterations are accompanied by major functional changes. The progressive peritoneal collagen deposition and fibrosis reduce the osmotic conductance of glucose and restrict water transport, ultimately leading to a loss of free-water transport and UFF [3,21]. Assessment of free-water transport across aquaporin-1 and the determination of sodium sieving provide significant information on membrane properties. An early and disproportionate decrease of osmotic conductance, as well as a decreased sodium sieving, may be a prognostic factor of EPS. Early changes in water transport capacity reflect significant structural and molecular alterations in the PM of patients who subsequently developed EPS [21]. This decrease in the free water transfer could be related to the degree of interstitial fibrosis and thus the collagen content in the PM.

Several studies suggest that another important step to EPS is PD related, namely peritonitis episodes [10,13,30,33], particularly if caused by *Staphylococcus aureus*, *Pseudomonas*, and/or fungi species [26,56]. In a subset of patients, prolonged, smoldering inflammation following peritonitis may particularly accelerate the peritoneal transformation process [57]. The PD catheter itself can trigger inflammation, either directly as a foreign material [58,59] or by acting as a site of bacterial biofilm formation [60]. Following dialysis catheter removal for refractory bacterial peritonitis, patients with persistent sterile peritoneal inflammation have a 31% likelihood of developing full spectrum EPS associated with a mortality rate of 36% [61]. Other factors associated with EPS development are a high peritoneal transport status [41] requiring high dialysate glucose concentrations to achieve sufficient UF and possibly icodextrin use in long-term PD patients [12,62,63]. EPS could also be idiopathic or a para-malignant phenomenon [8,64] and be driven by genetic predisposition [41,65,66]. Polymorphisms in genes expressing inflammatory [67], angiogenic [68], and fibrotic [67,69] factors may influence an individual’s susceptibility to EPS development, and at least in part explain geographic variations in EPS rates [18]. Other contributors are certain disinfectants. In 1991, Lo et al. reported severe EPS in 4 out of 18 CAPD patients using chlorhexidine in alcohol for the connection procedure [70], and in 1997, Keating et al. reported two cases of EPS following elective colorectal surgery and peritoneal lavage with an aqueous povidone iodine solution [71]. Early reports suggested that a beta blocker, such as practolol, induces peritoneal sclerosis via inhibition of peritoneal surfactant release; however, this is not reconfirmed for frequently used beta blockers, such as propranolol [72,73].

Calcineurin inhibitors (CNIs) like tacrolimus and cyclosporin have a profibrotic effect through the up-regulation of TGF-β1 and other profibrogenic factors, and may promote peritoneal matrix accumulation [36,74]. In experimental rat models, cyclosporin combined with prolonged peritoneal exposure to dialysis solutions was related to increased peritoneal fibrosis and angiogenesis [75]. EPS incidence is relatively high shortly after renal transplantation [76,77], possibly due to the acute cessation of PD that is accompanied by a loss of the peritoneal rinsing effect and due to the profibrotic effect of immunosuppressive medication like CNIs [7]. In a Japanese registry with 1958 PD patients prospectively followed over 4 years, EPS occurred in 48 patients, where two thirds of the cases occurred after renal transplantation (RTx) [6].

At present, the relative contribution of each of these factors to the development of EPS is not well described and may vary considerably in individual patients. Overall, long-term PD treatment and peritonitis episodes appear to play a predominant role. In a competing risk analysis in 17,300 PD patients, a long PD duration, younger age, and a primary renal disease was considered to have a low risk of death as compared to all other diseases (polycystic disease, isolated glomerulonephritis, and chronic pyelonephritis), and strongly predicted the risk of EPS, while diabetes and gender did not. Other studies also demonstrated an increased incidence of EPS in younger patients, which is further enhanced with the duration of PD [11,12]. None of these analyses could comprehensively assess the spectrum of potential factors contributing to the development of EPS. The “two-hit hypothesis,” postulates that one or more of the above-mentioned factors could serve as a second hit on top of a long-term duration of PD (first hit) and may act as a trigger for EPS [40,41]. The first hit is the disruption of the natural peritoneal and mesothelial physiology via the chronic exposure to toxic PD fluids, which induces modifications that can be evident in any patient on long-term PD. In a small number of patients, a “second hit,” such as a major inflammatory stimulus superimposed on the chronically injured peritoneum, induces the transformation to EPS.

### 3.2. Risk Factors

Integrating the data regarding pathophysiology and the clinical observations, the risk factors for developing EPS are several but the predominant ones are the duration of PD treatment and the episodes of peritonitis during the course of the treatment [10,13,19,20,21,22,23,24,25,26,27,28,29,30,33]. PD duration as a risk factor involves the constant and chronic effects that the PD fluid constituents (high glucose, lactate, low pH) have on the PM along with the mechanical effects on the PM by the fluid introduction into the cavity. In this context, in cases of high transporter PD patients where high glucose concentrations are required in order to achieve adequate ultrafiltration the EPS risk increases [16,41,62,63]. Renal transplantation accompanied by the acute cessation of PD and concomitant immunosuppression increases the incidence of EPS and this may be relevant to the abrupt lack of the mechanical effects of the PD fluid on the PM [7,76,77]. Peritonitis events in PD patients, especially induced by *Staphylococcus aureus, Pseudomonas*, and/or fungi species, accelerate the deterioration of the membrane even after the resolution of the peritonitis in some cases [10,13,26,30,33,56]. Other risk factors that may promote EPS are certain disinfectants (e.g., chlorhexidine, povidone iodine) or some beta blockers (e.g., practolol) [70,71,72,73]. Finally, certain polymorphisms in genes involved in inflammation, angiogenesis, and fibrosis may increase the susceptibility of a PD patient toward developing EPS [18,67,68,69].

## 4. Diagnosis of EPS

Clinical symptoms of EPS comprise irregular and relentless or frequent complaints of gastrointestinal obstruction with severe abdominal pain, nausea, and vomiting. The inability to maintain adequate nutrition leads to weight loss, and in some patients, the need for parenteral nutrition [9]. Ultrafiltration is often severely compromised and eventually lost. The transporter status is high in peritoneal equilibration tests, but these findings are not mandatory. In the 2007 U.K. survey, 7 out of 63 patients with EPS were low average transporters, 27 out of 71 had more than 1 L of ultrafiltration per 24 h, and a few had significant residual renal function [9]. Macroscopic inspection and/or radiological studies yield evidence of peritoneal sclerosis, calcified peritoneal thickening, and encapsulation of the intestine [9]. CT scans reveal a thickened peritoneal membrane extending from the visceral to the parietal peritoneal surface, bowel tethering, localized or diffuse peritoneal calcifications, and encasement of the small bowel that limits normal motility, a condition which is often referred to as abdominal “cocooning.” The thickened fibrotic membrane creates cystic fluid collections that underline the diagnosis of EPS. As EPS develops, patients may suffer from subclinical bowel ischemia, translocation of bacteria across the intestinal wall into the peritoneal cavity, and exacerbation of inflammation, fibrosis, and sclerosis [41,78,79]. Four stages of EPS have been classified by Nakamoto et al. using abdominal symptoms and inflammation, encapsulation, and intestinal findings [79]: stage 1 (pre-EPS), stage 2 (inflammatory), stage 3 (encapsulating), and stage 4 (chronic) [80]. More specifically, the clinical findings in stage 1 include ultrafiltration deterioration, as seen by an increase in the transport rate, low blood protein levels, ascites, bloody dialysate, and peritoneal calcification. In stage 2, there are increased markers of inflammation (white blood cell counts and C reactive protein) accompanied by fever, ascites, bloody dialysate, and a loss of appetite and weight. In stage 3, the resolution of inflammation is accompanied by the clinical signs of ileus, while in stage 4, there is complete ileus accompanied by an abdominal mass and anorexia. In view of the severe consequences of EPS, the threshold should be low for investigations and comprise thorough clinical investigations, peritoneal function tests, and radiological studies, i.e., plain X-ray for calcifications, CT scan, MRI, and eventually laparoscopic inspection of the peritoneum, with a tissue biopsy to provide a definite diagnosis [81]. The serum beta 2 microglobulin (beta2MG) has been suggested as an independent predictor of EPS [82]. This multiple regression analysis in 25 EPS and 25 age- and dialysis-vintage-matched patients included primary disease, serum urea nitrogen, creatinine, beta2MG, C-reactive protein (CRP), and the peritoneal equilibration test (PET) transporter status. However, the odds ratio was 1.2 with a sensitivity of 64% and specificity of 80% at a cut-off level of 37 mg/dL, while a slightly lower cut-off (30 mg/dL) yielded an odds ratio of 1, thus a direct pathophysiological link is questionable.

## 5. Treatment of EPS

Unfortunately, there are no guidelines or standard treatments for EPS. Solid scientific evidence is lacking and the assessment of the various treatments of EPS is complex and based merely on observational reports. A publication bias favoring positive treatment outcomes is likely. Targeted pharmacological therapies are missing and prospective trials with sufficient patient numbers are difficult to realize. Still, there is some empiric knowledge, which may guide treatment strategies; however, there is limited and only observational evidence regarding therapy [8,25,83,84]. Therefore, based on the available evidence, the early treatment of EPS is justified and based on glucocorticoids, tamoxifen, and nutritional support, while immunosuppression alone may not be effective. Still, all pharmacological treatments of EPS need to be accompanied by therapy of the clinical symptoms, such as pain medication and parenteral nutrition, in the case of intestinal obstruction. In most patients developing EPS while on PD, PD has been discontinued, but some rinsing of the peritoneal cavity may be beneficial. An overview of the currently applied and experimental treatments for EPS is given in Table 1.

### 5.1. Glucocorticosteroids (GC)

GC use for the treatment of EPS is reported in many observational reports with varying formulation, dosing, duration, and treatment end points. First-line use appears obvious in the management of EPS [9,25,83,84]. GC act by suppressing inflammation, preventing fibrin deposition and collagen synthesis and maturation [85], while thickening of the peritoneal membrane decreases and may even disappear [8]. Steroids may also prevent intraperitoneal fluid accumulation and the formation of ascites [86,87]. GC inhibit the glucose–mediated induction of monocyte chemoattractant protein-1 (MCP-1), which is crucial in recruiting monocytes and promoting fibrosis in peritoneal sclerosis.

Mori et al. reported on the successful use of GC alone in a patient with EPS [87], while Martins et al. reported on one patient who was successfully treated for EPS with prednisone in combination with azathioprine [88]. Dejagere et al. stressed the importance of high-dose GC therapy based on a successful treatment of EPS occurring in a patient early after RTx and of a subsequent EPS relapse with an increased dose of the GC [89]. Kuriyama et al. reported on six patients not treated with GC before 1997, who all died within 8 months, while the five patients treated with 0.5 mg/kg prednisolone all survived [90].

Kawanishi et al. reported that only 38.5% of patients recuperated on steroids while the remaining passed away or required surgical intervention [6]. Likewise, the Pan-Thames EPS study, the largest retrospective study involving steroids, found no improvement in median survival. However, treatment groups were too heterogeneous for a significant analysis [77]. In a summary of papers and abstracts on the effectiveness of GC for EPS in Japan, 15 patients had stage II EPS (nausea and diarrhea, mild inflammation, partial encapsulation, and intestinal swelling), 20 patients had stage III (ileus mild to severe inflammation, major encapsulation, and adhesion), and 4 patients had stage IV EPS (chronic ileus, no to mild inflammations, shrinkage of intestinal loops). Mean PD duration was 102 months (range: 4–192 months), daily GC dose ranged from 2.5 to 60 mg, given over 1–36 months. Recovery of bowel function was achieved in 79% of stage II cases, in 57% of stage III cases, and in 50% of stage IV cases, mortality rates were 3.6, 14.3, and 25%, respectively [91]. These data demonstrate the efficiency of GC treatment, especially when started in the early stages of EPS (with ascites only and mild inflammation) [80,90,92]. GC pulse therapy has been suggested during the early stage of EPS [93].

GC dosing and treatment duration are not well established. Some authors recommend three initial methylprednisolone bolus doses of 500–1000 mg per day and then 0.5 to 1 mg/kg/day for one month followed by dose tapering with a total treatment duration of 12 months [8,11,87,89,90]. Others recommend a dose of 0.5–1 mg/kg/day of prednisolone for 2–4 weeks and then slow tapering over months depending on clinical symptoms and signs of inflammation [88,89,93]. 

### 5.2. Non-Steroidal Immunosuppressants

Azathioprine, rapamycin, mycophenolate mofetil, sirolimus, and cyclosporine have been used to treat EPS, either alone or in combination with steroids [94,95,96,97,98,99,100], and may improve EPS, at least in the case of ongoing inflammation [8]. The evidence regarding the effectiveness is based on case reports only, i.e., clinical trials are lacking [85]. The fact that EPS frequently develops after RTx, i.e., in patients already receiving GC, argues against a major therapeutic benefit of drugs such as CNI, mycophenolate, and azathioprine. However, this notion has to be balanced against the fact that the rinsing effect of PD is lacking after RTx and that CNI are co-administered in most cases, which are known to promote fibrosis. Retrospective studies and registry data suggest that after 10 years on PD, a transplant recipient has a 10% risk of EPS on conventional CNI regimens versus a 1% risk on low dose CNI + mTOR inhibitors. Adaptation of the immunosuppressive protocol toward mTOR inhibitors, MMF, and corticosteroids may be justified in order to prevent post-transplant EPS in patients at high risk [101].

#### 5.2.1. mTOR Inhibitors

Sirolimus and everolimus are the mammalian target of rapamycin (mTOR) inhibitors. Based on their antiproliferative action, they may be an effective treatment for EPS in combination with GC [100,102,103]. In rat models, they have been shown to reduce peritoneal thickness, vascularization, and fibrosis [103,104] through molecular mechanisms, including the induction of E-cadherin, inhibiting EMT, and suppression of a-smooth muscle actin expression (a-SMA) [100,105].

Minetto Brabo et al. reported a successful EPS treatment with sirolimus combined with a GC [100]. Two case reports suggest favorable effects of everolimus [106,107]. On the other hand, two cases of post-RTx EPS have been reported despite sirolimus [108] and everolimus use [109].

#### 5.2.2. Mycophenolate Mofetil

Mycophenolate mofetil (MMF) is widely used for maintenance immunosuppression in renal transplantation and inhibits inosine monophosphate dehydrogenase reversibly and highly selectively. Also, it inhibits intimal hyperplasia and attenuates the expression of genes favoring smooth muscle cell proliferation and migration [110]. Recent studies have shown that MMF also has antifibrotic effects. In rat models, MMF has led to a significant reduction of peritoneal thickness, inflammation, and the fibrosis score [111,112]. LaFrance et al. reported on three patients who were successfully treated with a combination of MMF and corticosteroids [96].

#### 5.2.3. Azathioprine

Azathioprine (AZA) is an immunosuppressive antimetabolite with anti-inflammatory effects. In rats with chlorhexidine-induced EPS, where GC reduced the degree of fibrosis, whereas AZA did not improve any histological parameter [85]. Wong et al. published a successful case with AZA and corticosteroids [95]. Martin et al. reported recovery of a severe case of EPS with the resumption of oral nutrition after 10 days of AZA and GC [88].

## 6. Other Therapies

### 6.1. Tamoxifen

Tamoxifen is a selective estrogen receptor modulator, which acts as a nonsteroidal estrogen antagonist in many tissues and is used in females with breast cancer. Furthermore, tamoxifen has been used for treating retroperitoneal fibrosis, fibrosing mediastinitis, idiopathic sclerosing cervicitis, Dupuytren’s palmar fascia, and rapidly growing desmoid tumors. These approaches take advantage of the tamoxifen-induced TGF-β1 suppression of fibroblasts [113,114]. Since TGF-β1 is essentially involved in PD related peritoneal membrane transformation, i.e., in the process of peritoneal fibrosis and MMT, tamoxifen should also mitigate the key pathomechanisms of peritoneal damage. Tamoxifen may also reverse TGF-β1-mediated suppression of matrix metalloproteinase-9 (MMP9) and thus restore type IV collagen and collagen degradation and favor mesothelial healing [115].

Loureiro et al. demonstrated reversal of TGF-β1-induced MMT of mesothelial cells (MCs) in vitro by and partially of the mesenchymal phenotype of effluent-derived MCs [116]. Another possible anti-fibrotic mechanism associated with tamoxifen is the down-regulation of the pro-fibrotic connective tissue growth factor [117]. Next to the anti-MMT and anti-fibrotic effect, tamoxifen has anti-angiogenic properties activities and preserves peritoneal membrane integrity [116]. Thus, tamoxifen serves as an alternative to GC in the treatment of EPS with fewer and more tolerable side effects. 

Tamoxifen use in PD patients with EPS was first successfully described in 1992 [118]. Since then, several anecdotal reports describe favorable outcomes with tamoxifen, either alone or in combination with corticosteroids or immunosuppressants [114,119,120,121,122,123,124,125,126,127,128,129]. A Dutch multicenter retrospective study demonstrated a significantly lower mortality rate in 24 tamoxifen-treated EPS patients (46%) as compared to 39 non-tamoxifen-treated EPS patients (74%). Survival, adjusted for age, year of diagnosis, use of corticosteroids, presence of functioning transplantation, use of parental nutrition, and center influences, was longer in tamoxifen-treated patients (HR 0.39, *p* = 0.056) [130]. On the other hand, a questionnaire sent to 11 PD centers in the U.K. in 2007 identified 111 EPS patients with a 53% mortality rate after PD discontinuation. No advantage of tamoxifen, immunosuppression, both, or no treatment could be delineated, but treatment may have been driven by the severity of the disease [77]. Summers et al. reported a heterogeneous group of 27 EPS patients, of which, 16 with severe EPS underwent surgery. Five of these 16 patients received tamoxifen and 2 died, while 8 received no specific medicinal therapy and 5 of them died [120]. Del Peso et al. reported on 23 patients with peritoneal sclerosis who had not yet developed EPS. Nine were given tamoxifen and none developed EPS, but abdominal complications improved. Of the other 14 patients who did not receive tamoxifen, 4 developed EPS [121]. Tamoxifen may therefore be particularly useful during the prodromal stage at preventing full-blown EPS [131].

Tamoxifen is generally well-tolerated. Potential side effects include hot flushes, nausea, fatigue, endometrial carcinoma, ischemic stroke, pulmonary embolism, and deep venous thrombosis. However, Tsai et al. and Korzets et al. have suggested that tamoxifen should not be used in patients with both EPS and calciphylaxis due to the promotion of a hypercoagulable state [132,133]. In addition, tamoxifen should be administered with caution to patients with lupus nephritis and EPS, also having in mind their hypercoagulable state.

Taken together, there is observational evidence suggesting the beneficial effects of tamoxifen in the treatment of EPS. While GC and immunosuppressants treat the inflammatory components of EPS, tamoxifen may act via antifibrotic and anti-angiogenic effects. Due to the relatively good clinical tolerability, it may be started in cases of imminent or early stages of EPS, i.e., to prevent the development of the full clinical picture of life-threatening EPS and in cases with uncertainty about the underlying inflammatory component of EPS, e.g., in histological findings with predominant fibrosis. Of note, up to now, tamoxifen has mostly been administered in combination with steroids [8].

### 6.2. Renin–Angiotensin–Aldosterone System (RAAS) Inhibition 

At present, it is unclear to what extent fibrogenic and inflammatory effects of the RAAS are involved in the pathogenesis of EPS. Angiotensin II has pro-inflammatory and pro-fibrotic effects that act by stimulating the TGF-β1 production induced by the high glucose content of the dialysate [134]. The anti-fibrotic properties of RAAS inhibition slows renal fibrosis and progression of renal disease, and according to retrospective observational data, may attenuate the loss of residual renal function, even in patients on PD [135].

In an in vitro human peritoneal mesothelial cell model, perindopril and candesartan reduced TGF-β1 synthesis and high-glucose-induced cell proliferation [134]. Rat models of PD demonstrated less peritoneal fibrosis and angiogenesis with a RAAS blockade [42,136]. In hypertensive rats with EPS induced by a glucose-containing acidic PD solution, oral administration of the angiotensin II (AII) receptor blocker (ARB) olmesartan, but not amlodipine, prevented the progression of peritoneal fibrosis and adhesions [137].

In patients on PD, angiotensin converting enzyme (ACE) inhibitors seem to have a positive effect on peritoneal function and seem to preserve the morphology of PM in long-term PD patients [58,135]. These studies suggest that RAAS inhibition may play a role in preventing EPS in PD patients. Their role is likely to be small as compared to key pathomechanisms of the PD fluid exerted chronic toxicity and inflammatory insults induced by severe peritonitis episodes. Duration of the ACEi/ARB treatment did not differ between PD patients who developed EPS and time-matched controls [138]. Further studies are needed before a RAAS blockade should routinely be performed in PD patients to preserve residual renal function to mitigate peritoneal membrane transformation and to prevent the development of EPS. 

### 6.3. Surgical Treatment

In severe stages of EPS, surgical enterolysis may be effective to treat intestinal obstruction and to remove the inflammatory tissue that may otherwise gradually transform into fibrotic and sclerotic tissue. The presence or absence of peritoneal calcification may be important factors for determining the indication for surgical treatment is necessary [93]. On the other hand, a large adhesiolysis may induce further trauma and inflammation, potentially aggravation EPS and creating additional adhesions and constriction [139]. Surgery can reverse the bowel obstruction but relapses may occur. Thus, surgery is rather indicated for patients suffering from EPS with severe symptoms of bowel obstruction [140]. Others suggested that surgery should be performed after inflammation has subsided [93]. At present, there is not sufficient data supporting this notion. Surgical measures should be taken with caution and performed by experienced teams [139].

## 7. Experimental Approaches to EPS Treatment

### 7.1. N-Acetylcysteine

N-Acetylcysteine (NAC) is a licensed mucolytic drug and is also given in cases of paracetamol intoxication. NAC can scavenge reactive oxygen species and replenish antioxidant molecule levels in the body [141]. Supplementation of antioxidative compounds including NAC has been considered for dialysis patients since they suffer from high oxidative stress levels and exhaustion of antioxidative metabolites, but this has not yet been implemented in clinical routine [137]. In a rat model of peritoneal adhesion induced by a cecal abrasion, a single, concomitant administration of NAC significantly reduced inflammatory cell invasion, fibrosis, and adhesion formation [138]. In a rat model of chlorhexidine-induced EPS, NAC was more efficient than peritoneal rest with regard to a stronger reduction in peritoneal inflammation and vascularization, while the degree of fibrosis was comparable [136].

### 7.2. Colchicine

Colchicine has an anti-inflammatory and antifibrotic action and has been applied for fibrosing diseases, such as liver fibrosis, as it suppresses TGF-β1 transcription [140]. Similar to NAC, colchicine provided beneficial effects in the rat model of chlorhexidine-induced EPS, with a superior reduction of peritoneal inflammation and hyper-vascularization than peritoneal rest [142].

### 7.3. Pentoxifylline

Pentoxifylline (PTX) is a methylxanthine compound with immunomodulatory and antifibrotic properties. PTX may decrease intra-abdominal adhesion formation via increasing peritoneal fibrinolytic activity, suppressing angiogenesis, decreasing collagen synthesis, and reducing peritoneal fibrosis. In an animal model, intra-abdominal adhesions were markedly reduced by PTX treatment [143]. In a rat model of high-glucose-induced peritoneal damage, PTX reduced peritoneal thickening and collagen expression; high-glucose-induced proinflammatory interleukin-6 (IL-6), MCP-1, and TGF-β1 were reduced; and the ultrafiltration rate was preserved [144].

### 7.4. Rosiglitazone

Rosiglitazone is a peroxisome proliferator-activated receptor (PPAR) agonist. PPARs are the main controllers of key metabolic pathways of a range of inflammatory responses in fibrosis progression. PPAR gamma receptors are expressed in mesothelial cells [145]. PPAR gamma plays a significant role in cell differentiation, as well as in anti-inflammatory and antiangiogenic responses. They might therefore have a potential role in peritoneal defense. In a rat model of EPS, rosiglitazone was more effective than peritoneal rest regarding the mitigation of peritoneal hypervascularization and fibrosis [146].

### 7.5. Pirfenidone

Pirfenidone has antifibrotic and anti-inflammatory properties. In a rat model of peritoneal adhesion, pirfenidone reduced tissue inhibitor of metalloproteinases-1 (TIMP-1), tumor necrosis factor-α (TNF-α), and TGF-β1 protein abundance; increased MMP-9; and reduced abdominal adhesions, and may therefore also be useful in the context of EPS [147].

### 7.6. Thalidomide

Thalidomide was originally licensed as a sedative and anti-emetic. It inhibits basic fibroblast growth factor and VEGF effects and exerts immunomodulatory, anti-angiogenic, anti-proliferative, and antifibrotic actions [148]. It is used for the treatment of various inflammatory and autoimmune diseases [149]. In a mouse model of chlorhexidine-induced peritoneal damage thalidomide co-treatment significantly ameliorated submesothelial thickening and angiogenesis, decreased numbers of proliferating cell nuclear antigen (PCNA)- and VEGF-expressing cells, myofibroblasts, and TGF-β-positive cells [148]. Similar benefits of thalidomide were described in a rat model of chlorhexidine induced peritoneal damage with reduced fibrosis; a rise in myeloperoxidase activity; a reduction in tissue TNF-α, IL-1β, TGF-β, and VEGF in nitrotyrosine and nuclear factor κB activation [150].

### 7.7. Itraconazole

Itraconazole is an anti-fungal agent. Lately, it has been recognized as an inhibitor of the sonic hedgehog signaling pathway, which is involved in fibrogenesis. In a mouse model of chlorhexidine induced peritoneal damage, itraconazole significantly reduced the sonic hedgehog signaling pathway, TGF-β1, and α-smooth muscle actin expression; decreased TGF-β1 expression; and reduced peritoneal fibrosis to one third of chlorhexidine-only treated mice [151].

### 7.8. Dissolved Molecular Hydrogen (H_2_)

H_2_ exerts anti-oxidative and anti-inflammatory effects [152]. In a less aggressive model of peritoneal damage than chlorhexidine administration, i.e., single injection of a neutral pH PD solution with low GDP content over 10 days in rats, the addition of H_2_ mitigated submesothelial cell infiltration, mesothelial cell proliferation and apoptosis, and vimentin positivity, with the latter suggesting less MMT, but this could not be reconfirmed by other MMT markers [153]. Following an unsuccessful attempt to treat full-blown, histologically proven EPS with prednisolone for 30 days, Terawaki et al. added dissolved H_2_ to the hemodialysate, and 10 days later, also to the peritoneal lavage fluid. The patient’s symptoms steadily improved. At day 60, the catheter was removed and another peritoneal biopsy was taken. The latter revealed a normal peritoneal thickness and an intact mesothelial layer. The patient remained free of clinical EPS signs during the subsequent 18 months of observation [154]. 

### 7.9. Peritoneal Stem Cell Treatment

A number of experimental studies on stem cell therapy to reduce PD-induced peritoneal damage have been accomplished, of which 11 were included in a recent systematic review [155]. Ten of these studies used mesenchymal cells, while 7 chose an intraperitoneal injection route. Following various peritoneal insults, a stem cell treatment improved mesothelial integrity (100%), submesothelial thickness (100%) and fibrosis (86%), inflammation (63%), and angiogenesis (60% of the studies). Peritoneal transport function improved in all studies, i.e., the ultrafiltration rate increased and solute transport was reduced. In a first phase 1 clinical trial, Alatab et al. intravenously infused autologous adipose tissue-derived mesenchymal stem cells in 9 patients on PD for 77 (24–127) months, of which 6 had a high average and 3 a high transporter status on PET. No severe adverse events were reported; 14 adverse events were reported by 6 patients, mostly related to the liposuction procedure. During the 6 months follow up period, the daily UF rate increased, while the 24-h urine volume was unchanged. The dialysate/plasma (D/P) ratio for creatinine decreased, while urea and glucose transport remained unaltered; however, details on the PD regimen over time, including peritoneal glucose exposure, are not given. Other organ functions were not studied systematically [156].

## 8. Prognosis

The mortality of patients with EPS is very high, varying between 26–58%, and increases with PD vintage [77,157]. Most common causes of death due to EPS are malnutrition and sepsis. In the European Survey, 3 out of 22 children (14%) had died 4.8 (1.3–8.7) years after EPS diagnosis. Early diagnosis and timely treatment appear crucial [78]. However, great uncertainty exists regarding the best approach; discontinuation of PD, possibly with maintaining some rinsing procedure; timely treatment with GC and eventually tamoxifen, and the limitation of surgical approaches to severe cases of intestinal obstruction appears appropriate and may help improving prognosis. 

## 9. Conclusions

Despite the increased awareness of EPS over the last decade, EPS remains a much-feared complication of long-term PD. Several causes have been reported of which PD vintage, i.e., chronic exposure to unphysiological dialysis solutions over extended periods of time, especially when high glucose concentrations are used, and peritonitis episodes play a key role [37,158]. Together with several other insults, they trigger a network of pathomechanisms, which at some point cannot be counteracted by the peritoneum and result in massive, life-threatening peritoneal inflammation, fibrosis, and sclerosis. Regular and rigorous consideration of signs of incipient EPS is warranted. These are a predisposing high transporter status on PET, loss of peritoneal water transport, and declining UF, as well as otherwise unexplained abdominal pain, together with a reduced food intake. There is no consensus about the treatment of EPS. Given the present available data on EPS, early treatment is justified and based on glucocorticoids, tamoxifen, and nutritional support. Immunosuppression alone may not be effective. Multiple case reports have been published reporting successful treatment of EPS with a drug combination [100]. The accumulating clinical experience with tamoxifen is encouraging and suggests that EPS, previously considered a devastating consequence of PD with a high mortality rate, may be a manageable complication with an improving prognosis [122]. On the other hand, surgery (peritonectomy and enterolysis) should be considered in advanced cases with severe bowel obstruction. Whether neutral-pH PD fluids with low GDP content reduce the incidence of EPS is uncertain. While reports from Japan suggest less peritoneal transformation with these PD fluids, others do not [3]. The PD regime and dietary means should be optimized to reduce peritoneal glucose exposure, and in the case of imminent EPS, a timely switch to hemodialysis or (living related) transplantation should be considered, though a considerable number of EPS cases develop after PD discontinuation. Several novel and promising therapeutic approaches for EPS are on the horizon but are still far from being implemented in clinical routine. Therefore, in the case of EPS developing during PD, PD discontinuation is suggested, and a daily rinsing procedure may be considered with a fill volume as tolerated by the patient to mitigate the local inflammation and adhesions.

## Figures and Tables

**Table 1:** Overview on currently applied and experimental pharmacological treatments in EPS.

Class	Drug Name	Mode of Action
Glucocorticosteroids	Prednisone	Immunosuppressant, inhibits monocyte chemoattractant protein 1 (MCP-1) synthesis, regulates extracellular matrix (ECM) protein synthesis, ECM protein maturation
	Prednisolone	
Immuno-suppressants	Azathioprene	Inhibits DNA/RNA synthesis
	Rapamycin/Sirolimus	Inhibits T-cell/B-cell activation
	Mycophenolate mofetil	De-novo purine synthesis blockade
	Cyclosporine	Lowered T-cell activity
Hormonal antagonist	Tamoxifen	Blocks transforming growth factor-β1 (TGF-β1) signaling
	Angiotensin converting enzyme inhibitor (ACEi)/Angiotensin II receptor blocker (ARB)	Blocks TGF-β1 signaling
	Perindopril	Blocks TGF-β1 signaling, lowered cell proliferation
	Candesartan	
Mucolytic	Ν-acetylcysteine (NAC)	Reactive oxygen species scavenger
alkaloid	Colchicine	Blocks TGF-β1 mRNA expression
Xanthine derivative	Pentoxifylline	Fibrinoltyic, suppressed collagen synthesis, angiogenesis
Anti-diabetic	Rosiglitazone	Peroxisome Proliferator-Activated Receptor (PPAR)-agonist, suppressed inflammation, neovasculature
Anti-fibrotic, anti-inflammatory	Pirfenidone	Reduces tissue inhibitor of metalloproteinases-1 (TIMP-1), tumor necrosis factor-α (TNF-α), and TGF-β1 expression,
Immuno-modulator	Thalidomide	Anti-angiogenic, anti-proliferative, antifibrotic
Anti-fungal	Itraconazole	Decreased TGF-β1 expression
Autologous stem cell therapy		Mesothelial/submesothelial cellular layer repair

## Abbreviations

AGEs	Advanced Glycation End-products
a-SMA	a-Smooth Muscle Actin
AZA	Azathioprine
CNIs	Calcineurin Inhibitors
EMT	Epithelial-to-Mesenchymal Transition
eNOS	endothelial Nitric Oxide Synthase
EPS	Encapsulating Peritoneal Sclerosis
ESRD	End Stage Renal Disease
H_2_	Dissolved Molecular Hydrogen
HD	Hemodialysis
MCP-1	Monocyte Chemoattractant Protein-1
MCs	Mesothelial Cells
MMF	Mycophenolate Mofetil
MMP9	Matrix MetalloProteinase-9
MMT	Mesothelial-to-Mesenchymal Transition
mTOR	Mammalian Target of Rapamycin
NAC	N-Acetylcysteine
PD	Peritoneal Dialysis
PM	Peritoneal Membrane
PPAR	Peroxisome Proliferator-Activated Receptor
PTX	Pentoxifylline
RAAS	Renin–Angiotensin–Aldosterone System
TGF-β1	Transforming Growth Factor–β1
UF	UltraFiltration
UFF	UltraFiltration Failure
VEGF	Vascular Endothelial Growth Factor
